# An Attempt to Use Virtual Reality as a Tool to Reduce Patient Anxiety During Dental Treatment

**DOI:** 10.3390/jcm13226832

**Published:** 2024-11-13

**Authors:** Anna Ledwoń, Paweł Dębski, Przemysław Jędrusik, Sylwia Mielcarska, Hanna Misiolek, Michał Meisner, Maria Łopacińska, Małgorzata Skucha-Nowak

**Affiliations:** 1 Department of Dental Propaedeutics, Faculty of Medicine in Zabrze, Medical University of Silesia, pl. Dworcowy 3, 41-800 Zabrze, Poland; mskucha-nowak@sum.edu.pl; 2 Department of Psychiatry, Faculty of Medical Sciences in Zabrze, Medical University of Silesia in Katowice, 40-055 Katowice, Poland; pdebski@sum.edu.pl; 3 Centre of Online Education and Educational Effects Analysis, Medical University of Silesia in Katowice, 40-055 Katowice, Poland; pjedrusik@sum.edu.pl; 4 Department of Medical and Molecular Biology, Faculty of Medical Sciences in Zabrze, Medical University of Silesia, 19 Jordana, 41-800 Zabrze, Poland; sylwiamielcarska@gmail.com; 5 Department of Anesthesiology and Critical Care, School of Medicine with the Division of Dentistry in Zabrze, Medical University of Silesia, 41-808 Zabrze, Poland; hmisiolek@sum.edu.pl; 6 Institute of Psychology, Faculty of Social Sciences and Humanities, Humanitas University in Sosnowiec, 41-200 Sosnowiec, Poland; michal.meisner@humanitas.edu.pl; 7 Institute of Dentistry and General Medicine, 8 Łabędzia Street, 40-534 Katowice, Poland

**Keywords:** dental fear, virtual reality, relaxation techniques, dental anxiety, innovative dentistry

## Abstract

**Background/Objectives:** Dental fear and anxiety are prevalent issues in both children and adult patients, often leading to avoidance of dental care and deterioration in overall health. While virtual reality (VR) has been increasingly studied as a distraction tool in pediatric dentistry, its effectiveness among adults remains under-researched. This study aims to evaluate the impact of VR distraction on reducing anxiety and physiological stress indicators in adult dental patients. **Methods**: The study was conducted in a private dental office in Dobrodzień, Poland, involving 90 adult dental patients divided into three groups: two experimental (VR1 and VR2) and one control (C), with 30 patients each. Patients in the VR1 group experienced VR distraction during the first visit, and the second visit was conventional; the VR2 group experienced this in reverse; and the control group underwent traditional treatment in both visits. Physiological parameters (heart rate, saturation, and stress) and psychological anxiety levels (MDAS and STAI-X1) were measured at three time points during each visit. **Results**: Significant reductions in heart rate and stress levels were observed after VR exposure compared to the control group. VR also demonstrated a significant effect in decreasing anxiety levels (based on STAI-X1) during the visit with VR distraction. No significant changes were found in the saturation levels across the groups. **Conclusions**: The use of VR as a distraction tool during dental procedures effectively reduces physiological stress and anxiety in adult patients, suggesting its potential as a valuable tool in managing dental anxiety. Further research is recommended to explore the long-term benefits and patient satisfaction with VR-based interventions in dental care.

## 1. Introduction

Dental fear and anxiety are some of the most serious problems among both children and adult patients. Dental anxiety is estimated to be between 5.7% and 46.8% in different populations of children [1] and about 5–15% of adults [2,3]. Unaddressed dental fear in childhood often progresses into adulthood. Dental fear in adults has a negative correlation with their oral-related quality of life (OH-QoL) [4]. Generally, anxiety is defined as a feeling of worry, nervousness, or unease about something with an uncertain outcome [5]. According to a review from 2007, dental fear is described as a normal emotional reaction to various stimuli in the dental setting; otherwise, dental anxiety is referred to as a concern that something terrible is going to happen in connection with dental treatment; and dental phobia represents a severe subset of dental anxiety [6,7]. Dental fear and anxiety are some of the most common reasons for skipping dental appointments apart from financial concerns [8]. It can lead not only to poor oral health but also to deterioration of general well-being [9], by affecting cardiovascular or immune systems [10] and influencing metabolic disorders [11,12]. Fighting back dental fear and anxiety has been a challenge for dentists for many years. Various techniques have been used to achieve patients relaxation both prior to the visit and during one, for example: pharmacotherapy (e.g., nitrous oxide, triazolam, zolpidem, or midazolam [13]), music, audiovisual content, or relaxation techniques [14]. Those methods were thoroughly examined and numerous studies confirmed that they are effective. Nonetheless, patient expectations are changing and human perception is adapting quicky. Those are some of the reasons why new technologies are always in the field of interest when it comes to dental anxiety. The development of the IT sector allows us to adapt modern technologies for its use in dentistry. It is becoming more and more popular to use virtual reality (VR) to cope with dental fear and anxiety, especially among pediatric patients. In recent years, the potential use of VR in pediatric dentistry has become more frequently studied. However, there is a limited number of studies that test adults’ responses to VR distraction during a dental visit. There are some studies which analyzed how VR influenced patients with hypertension during dental extraction or what kind of audiovisual material in VR is more effective: nature or video games [15,16]. Virtual reality is a controlled, standardized environment, which can provide full immersion. The user is able to move around and interact with virtual environments, objects, and people [17]. The brain is so preoccupied with processing information presented via virtual reality that the patient has less attention available to process incoming pain signals [18]. The present study evaluates whether VR distraction can also be effective among adult dental patients. This study was created to verify both the objective, physiological response to VR (heart rate, saturation, and stress level) as well as the subjective opinion of it (surveys and psychological tests). The main aim of the study was to assess the effectiveness of virtual reality distraction among adult dental patients during dental treatment. The assessment was based on objective measurements: heart rate, saturation, and stress levels, and subjective self-reported questionnaires (MDAS, STAI-X1, and authors’ survey).

## 2. Materials and Methods

The present study was conducted in a private dental office in Dobrodzień, Poland. The design of the study was approved by the Bioethical Committee of the Medical University of Silesia in Katowice, Poland, with the acceptance date of 23 March 2022 (PCN/CBN/0052/KB/43/22). The experimental part of the study took part from November 2022 to September 2023. All dental procedures were performed by the same dentist.

### 2.1. Before the Study

Prior to the experimental study, a pilot study was performed. It was conducted to establish what kind of audiovisual material was the most suitable for adult dental patients. A short questionnaire, containing a huge variety of audiovisual materials to be embedded in virtual reality, was distributed among one hundred adult dental patients. The results showed that the most popular answers were related to nature: projection of landscapes, followed by interactive journeys, for example, a beach walk or mountain hike. Based on the results from the pilot study, projection of landscapes was chosen as the audiovisual content to be inserted into virtual reality during the experimental study. The pilot study is described in detail in another already published article [19].

Afterwards, open-license audiovisual materials from YouTube, featuring various landscapes accompanied by relaxing classical music were chosen (the video can be found at the following link: https://www.youtube.com/watch?v=klqvYbogLNY&list=PLLb6i1JpZ81BTy85aiosSuABgmEhmyzQg&index=7, accessed on 13 October 2024). A team of IT specialists from the Centre of Online Education and Educational Effects Analysis, Medical University of Silesia in Katowice, created an application suitable for virtual reality, presenting an apartment with a giant TV and a projection of landscapes on it.

### 2.2. Recruiting Patients

Ninety adult dental patients were recruited to participate in the study. The sample size calculation was based on the study protocol for a randomized controlled trial of similar design [20]. Patients who agreed to participate in the study were divided into 3 groups: 2 of those were experimental and the last one was a control group, with 30 patients in each group. They were chosen based on treatment needs and written informed consent was obtained for their participation in the study. Each patient visited the dental office twice, and both visits had to be of a similar duration (approximately 30 min) and at a similar advancement level, e.g., two first-class cavities in premolars treated conservatively or hygienization divided into the left and right sides. All visits were scheduled in the morning and early afternoon from 9 am. to 13 pm. The first group of patients (VR1) experienced VR on the first visit, while the second visit was conventional. The second group (VR2) had a conventional first visit, while during the second one, there was VR distraction applied. Finally, the control group (C) experienced traditional treatment on both visits. All the patients were randomly assigned to test and control groups, and the patients were not informed of which group they were assigned to. They did not know about VR distraction before the headset was presented to them directly before treatment. There was an allocation concealment. Before the dental procedure, the HADS questionnaire (Hospital Anxiety and Depression Scale) was used as a screening tool. HADS is a validated 14-item self-assessment on a 4-point Likert scale (range 0–3), developed by Zigmond and Snaith (1983). It is used to verify whether respondents are presenting states of anxiety and/or depression [21]. Those states could have affected the results of the study.

#### 2.2.1. Inclusion Criteria

- Written informed consent to participate in the study.
- Patients with treatment needs that matched the following criteria: a similar duration for both visits (approximately 30 min) and a similar advancement level of the dental procedure.
- Only adult patients over 18 years old were included.

#### 2.2.2. Exclusion Criteria

- Patients with severe hearing or visual impairment and/or mental disorders such as psychosis, post-traumatic stress disorder (PTSD), motion sickness, cybersickness, and previous history of epileptic seizures.
- Patients whose treatment needs had to be scheduled for more than 30 min.
- Patients with previous negative experiences with VR.
- Patients who required emergency dental care.
- Patients who scored ten points or more in HADS (Hospital Anxiety and Depression Scale) in the anxiety and/or depression section.

### 2.3. Equipment

In the present study, the Viveport Infinity Headset (HTC Corporation, Taoyuan City, Taiwan) paired with the laptop HP OMEN (HP Inc., Palo Alto, CA, USA) was used. The Unity3D engine (Unity 2021 LTS, version 2021.3.x) was used to run the VR application for landscape projection. Furthermore, the Medstorm Stress Detector (Medstorm Innovation AS, Oslo, Norway) paired with the laptop HP 7HP6570B (HP Inc., Palo Alto, CA, USA) was used to evaluate patients’ stress levels during the dental procedure, and a pulse oximeter (NOVAMA^®^, Warsaw, Poland) was used to assess the saturation (SAT) levels and heart rate (HR).

### 2.4. Experimental Procedure

#### 2.4.1. Before Procedure

Before the experimental procedure, patients who agreed to participate in the study were asked to complete two questionnaires in the dental office waiting room: the Modified Dental Anxiety Scale (MDAS) and Hospital Anxiety and Depression Scale (HADS). MDAS is a validated, self-complete screening instrument that allows us to assess the level of stress related to dental procedures as well as the presence of dental phobia. It is a 5-item questionnaire with a consistent answering scheme, with each item ranging from 1—“not anxious” to 5—“extremely anxious”. Afterwards, all answers were summed to construct a scale with a minimum score of 5 and a maximum of 25 [22]. Humphris et al. are the authors of the original English MDAS version (1995) [22], whereas the Polish adaptation was created by Dubielecka et al. The reliability of the Polish version of MDAS was rated 0.8 [23]. HADS is a validated 14-item self-assessment on a 4-point Likert scale (range 0–3), developed by Zigmond and Snaith (1983). It is used to verify whether respondents are presenting states of anxiety and/or depression. Its reliability ranks 0.74 for depression, and 0.85 for anxiety [24]. HADS scoring was calculated separately for the anxiety and depression parts of the scale. Results from 0 to 7 indicated that there was no emotional disorder. Between 8 and 10 points, respondents were in the risk group of anxiety and/or depression. Finally, 11 or more (up to 21 points) indicated that there was a great predisposition toward emotional disorders (anxiety and/or depression). It also allows us to evaluate valid measures of the severity of emotional disorders [21]. It was important for the study to exclude participants with the highest anxiety and depression scores, because their reactions to stress related to dental procedures may be disrupted by emotional disorders. HADS is not a tool to diagnose patients, but the higher the score, the bigger the risk that the patient presents with anxiety and/or depression disorders. Patients who scored above ten points on anxiety and/or depression (*n* = 10) dropped out of the further analysis. Both scales were used in their Polish versions.

#### 2.4.2. During Procedure

After completing both scales, patients were asked to enter the dental office. Each patient had a pulse oximeter placed on the index finger of their right hand and MedStorm Stress Detector electrodes were applied to their left palm. The first test group (*n* = 30)—VR1—had a VR headset fitted during the first visit. Patients were given approximately 3–5 min to adjust to virtual reality and a possibility to ask questions. During the whole dental procedure, patients were seated and measurements of heart rate, saturation, and stress levels were taken at three points of the survey: at the beginning—after a few minutes of adaptation, before initiation of dental procedure; in the middle of dental procedure—approximately 15 min from the start; at the end of the treatment (approximately 30 min from the start)—before the removal of the VR headset and before the patient was informed that the treatment had been completed.

The second visit for the VR1 group was traditional. The survey points remained the same. The second test group (*n* = 30)—VR2—had a conventional visit first, and then VR distraction was performed on the second visit. The control group (*n* = 30)—C—had both visits performed traditionally, with standard dental care.

Patients from the VR1 group had the VR headset presented directly before the first treatment procedure and they were not informed that the second visit would be without VR. Patients from VR2 had the conventional visit first without any information that during the second visit there would be VR distraction present. The control group was not informed that VR distraction was involved in the study. All participants were informed during the preoperative assessment that the study was created to evaluate stress levels among adult dental patients.

#### 2.4.3. After Procedure

After the required treatment, patients were asked to complete two other questionnaires: a psychological test—the State-Trait Anxiety Inventory, part one (STAI-X1)—and the authors’ questionnaire. STAI-X1 is a 20-item psychological test, created by Spielberger et al. (1983) [25,26]. Answers are rated on a 4-point scale (e.g., from “Almost Never” to “Almost Always”). Higher scores indicate greater anxiety. It is used to measure anxiety and helps to evaluate the respondent’s current emotional status. The STAI-X1 scale can be used to determine the actual intensity of anxiety induced by stressful procedures [26]—e.g., dental treatment. The second questionnaire was created by the authors and consisted of sociodemographic questions, as well as collecting information about patients’ feedback related to the dental procedures performed and general opinions about pain management in dentistry.

Both visits in the experimental (VR1, VR2) and control (C) groups were conducted as similarly as possible, to be comparable. Patients filled in questionnaires in the same sequence and the three survey points remained the same for both experimental groups. Both visits had a similar duration and advancement level.

### 2.5. Data Analysis

A Microsoft Excel database was created after finishing all the scheduled dental visits. Patients whose HADS score was higher than ten in anxiety and/or depression part were excluded from the study (*n* = 10). Afterwards, statistical analysis was performed using SPSS (version 29.0.2, IBM Corporation, Armonk, NY, USA).

Each group (VR1, VR2, and C) was analyzed according to the same sequence. Intra-group statistical comparison between heart rate (HR), saturation (SAT), stress, and time of the three survey points (0–15–30) were analyzed for both visits in each group, using univariate repeated measures ANOVA. Psychological factors (dental fear and anxiety) were also identified. A comparison of STAI and MDAS levels between the first and second visits was performed for each group, using a *t*-test for dependent samples. Furthermore, differences between the STAI in VR1/1—C/1 and VR2/2—C/2 were calculated using a *t*-test for independent samples.

Due to the substantial number of calculations and tests conducted using gathered data, it was decided that only the most statistically significant findings would be presented in results. The data gathered from patients who were randomly assigned to VR1 group (VR exposure during the first visit) were used to establish changes in psychophysiological parameters influenced by VR according to time (HR, SAT, and stress), whereas data from patients who were randomly assigned to VR2 group (VR exposure during the second visit) were used to analyze changes in psychological response (anxiety—STAI-X1), comparing a traditional dental visit to a dental visit with VR exposure. There were no statistically significant changes observed in dental fear, based on MDAS.

## 3. Results

The sociodemographic characteristics of the participants included in the study are shown in Table 1. For the results of the χ^2^ difference test for sociodemographic variables see Supplementary Materials.

In order to verify the differences between baseline MDAS values (before the first visit), univariate ANOVA was performed (1-ANOVA). There were no statistically significant differences between the MDAS values in VR1 vs. VR2 vs. the control (F(2,77) = 0.17, *p* > 0.05). Performed analysis proved that groups VR1, VR2, and C did not differ in terms of dental anxiety. The calculation was based on the Modify Dental Anxiety Scale (MDAS).

### 3.1. Psychophysiological Parameters (HR, Saturation, Stress)

**Figure 1:**
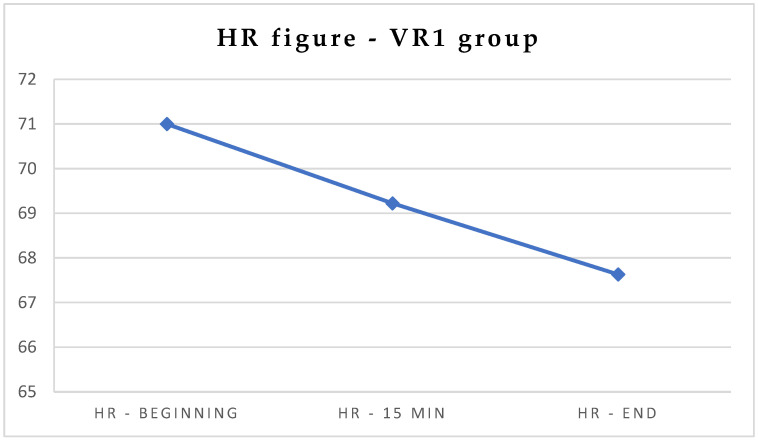
Differences in HR measurements in the VR1 group.

In order to describe how virtual reality affects psychophysiological parameters in adult dental patients, data from all VR1 participants were subjected to statistical analysis. Heart rate (HR), saturation (SAT), and stress at the three measuring points (at the beginning, after 15 min, and at the end of the procedure) were analyzed using univariate repeated measures ANOVA. The within-subject factor measured at the three levels (at the beginning, after 15 min, and at the end) was the time. The dependent variable was the HR or SAT measured using a pulse oximeter NOVAMA^®^ and the stress measured by a MedStorm StressDetector. This analysis revealed that the HR at the end of the study (M = 67.63; SD = 9.60) was significantly lower than the HR levels at the beginning of the study (M = 71.00; SD = 8.66). The significance of this difference was at *p* < 0.05. There were no significant differences in SAT or stress in the VR1 group (Table 2, Figure 1).

In comparison, the same univariate repeated measures ANOVA was performed for patients from the control group (C). Changes in HR, SAT, and stress were described according to the same measuring points (at the beginning, after 15 min, and at the end of the procedure). There were no significant changes in SAT and HR in the control group; however, there was a statistically significant increase in stress levels in the control group, from the beginning toward the end of the study (Table 3).

**Figure 2:**
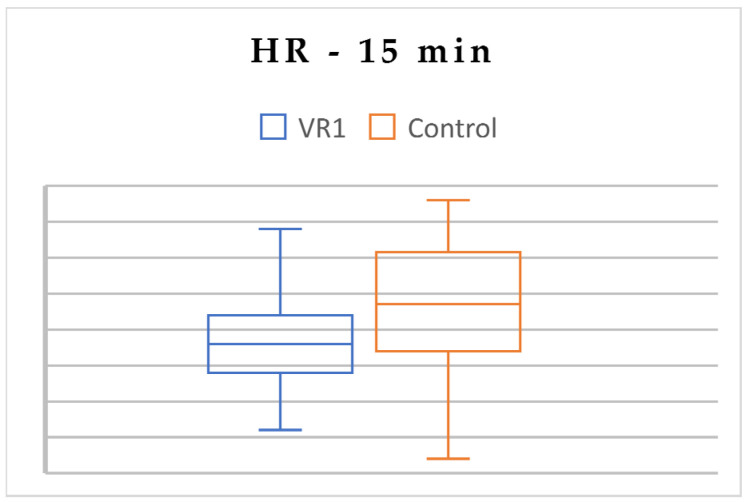
Differences in HR between VR1 and the control group, 15 min.

**Figure 3:**
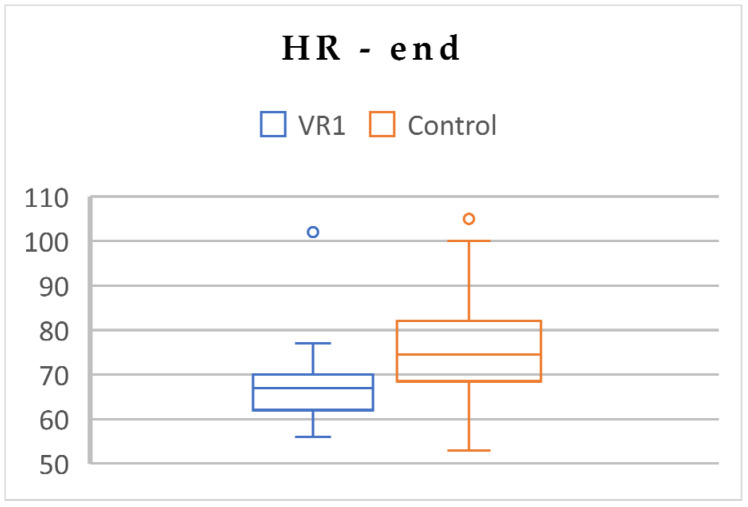
Differences in HR between VR1 and the control group, at the end.

**Figure 4:**
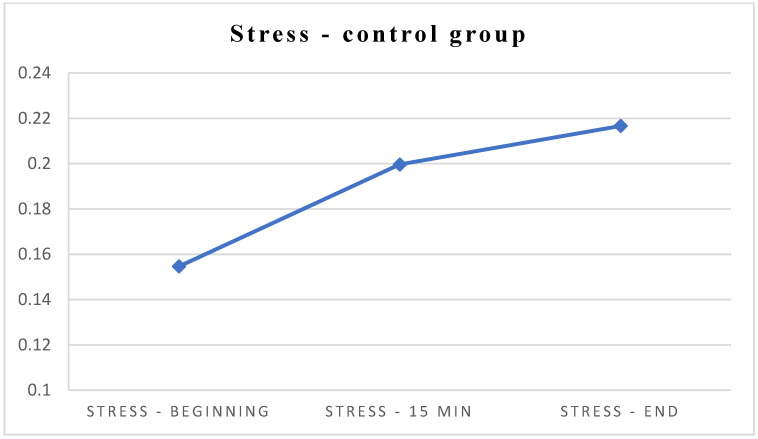
Differences in stress levels in the control group.

To assess the inter-group differences in psychophysiological parameters, an independent *t*-test was conducted for VR1 versus C at three time measuring points. At the beginning, there were no differences in the HR and stress levels between VR1 and C. Nevertheless, the saturation level was slightly higher in the VR1 group, but only at the beginning of the experimental procedure. Over time, after VR exposure, the HR and stress levels were significantly lower in VR1 group (Table 4; Figure 2, Figure 3 and Figure 4).

### 3.2. Psychological Parameters (STAI)

**Figure 5:**
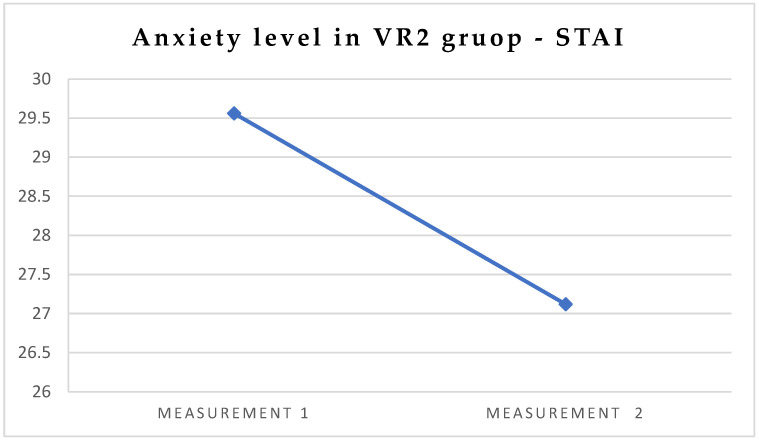
Differences in STAI, in the VR2 group, first vs. second visit.

In order to describe how virtual reality affects psychological parameters (anxiety) and how those parameters differ between traditional treatment (VR2/1) and VR-influenced visits (VR2/2), a dependent *t*-test was conducted based on STAI measurements. The test analysis showed that the average level of anxiety in the VR2/2 group (M = 27.12; SD = 5.41) was significantly lower compared to the level of anxiety in VR2/1 (M = 29.56; SD = 7.80), *t*(24) = 2.29; *p* < 0.05 (Table 5, Figure 5).

In comparison, to check the difference in the STAI levels between the two standard dental visits (C/1 and C/2), a dependent *t*-test analysis was performed. The test analysis showed that the average level of anxiety in the control group in C/2 was the same as in C/1 (*t*(27) = 1.09; *p* > 0.05) (Table 6).

To assess inter-group differences in psychological parameters (anxiety), an independent *t*-test was conducted for STAI measurements in VR2 versus C. There were no significant differences in the comparison of STAI between the VR2 and control groups (Table 7).

A statistically significant decrease in psychological parameters in adult dental patients in the VR2 group compared to a lack of difference in the control group may indicate that VR helps reduce anxiety during dental visits.

## 4. Discussion

Dental anxiety often leads to deterioration in oral health. Adult patients are as keen as children to avoid the required treatment because of fear related to dental visits. It became more and more popular over the years to search for distractions for dental patients to help them overcome dental anxiety. A friendly environment is crucial for patients to perceive dental visits as stressless. Virtual reality (VR) is a relatively new technology that creates a virtual environment and engages patients in an immersive, interactive world [27].

In the present study, adult dental patients experienced VR during dental visits. Most of the studies found in the literature were focused on the use of VR distraction in pediatric dentistry. However, there are some publications which described adults’ reaction to VR in dental offices. Gujjar et al. indicated that it can be beneficial for adult patients with dental phobia to experience stressful situations, e.g., dental examination or cavity preparation in a standardized, consistent VR environment [28]. Almugait et al. compared pain perception during local anesthesia deposition using either a topical anesthetic gel or VR distraction. Even though the results were statistically insignificant, patients reported less pain after experiencing VR distraction [29]. Usage of VR distraction in adult patients undergoing dental extractions proved VR to be beneficial for the majority of patients. A total of 87.5% of patients reported that they would like to experience VR distraction during their future visits [30]. When patients with hypertension were analyzed during tooth extraction with and without VR, it was proven that VR is a helpful tool in reducing blood pressure and heart rate during the dental procedure. This can be of great use, especially in patients with hypertension [15]. The audiovisual material which was chosen for the study was based on patients’ preferences established in a pilot study [19]. In the present study, the projection of landscapes accompanied by relaxing instrumental music was chosen. For adult dental patients, other researchers chose an underwater aquarium [30] or an animated short movie [29]. Yamashita et al. compared the effectiveness of nature videos with video games embedded in VR among adult dental patients. The results showed that both distraction methods were effective in reducing patients’ pain and anxiety, with slightly better results for video games [16]. In pediatric dentistry, a variety of animated movies were chosen, e.g., a VR roller coaster [31], SnowWorld [18], or cartoon like “Tom and Jerry” [32] or “SpongeBob” [33]. The levels of dental anxiety, psychological conditions, and post-procedure sensations were evaluated by a number of questionnaires. The Modified Dental Anxiety Scale (MDAS) was used to assess patients’ stress prior to the visit. It is a validated questionnaire helpful in establishing patients’ stress levels related to defined dental procedures. MDAS is a conventional tool for measuring dental anxiety used in adults patients [20,28,30]. Moreover, in the present study, the Hospital Anxiety and Depression Scale (HADS) was used to exclude patients with the highest probability of anxiety and depression disorders. To assess the effectiveness of VR distraction post-procedure, the self-reported psychological test STAI-X1 was used in the study. In order to evaluate the degree of pain or anxiety in adults, self-reported questionnaires are often used [20,28], whereas in pediatric patients, the most popular scales are the Wong–Baker Faces Pain Scale (WBFP) and Venham’s picture test (VPT), which are pictorial and therefore easier to carry out with children [33,34,35]. Patients’ physiological responses to stressful factors (dental treatment), heart rate and saturation, were measured by a finger pulse oximeter. The same protocol was commonly used in various studies with both children and adult patients [33,34,36]. Innovatively, in the present study, stress levels were also measured by a MedStorm Stress Detector. The device measured stress levels by monitoring skin conductance and converting electrical signals into numeric values. Electrodes were placed on patients’ palms according to producer’s instructions, and skin conductance was shown as a graph, updated every 2 s, on computer screens [37].

In general, the results showed that VR distraction can be beneficial for adult dental patients. Virtual reality exposure therapy proved to be effective in the treatment of dental phobia [28]. Ougradar et al. proved that patients had positive views on VR during extraction procedures and the vast majority of them would like to reuse it during future treatment procedures [30]. Even though the results of some comparisons were statistically insignificant, they presented desirable directions (with *p*-value higher than 0.05) in reducing dental anxiety. Alumgait et al. showed similar outcomes; desirable differences in results were observed, though not significant [29].

In the present study, feedback from patients who experienced VR distraction was mainly positive. The visit was described as interesting, shorter, and very pleasant. Patients reported that they felt relaxed and their attention was diverted from the dental office. The vast majority described VR as beneficial, and there were only a few who did not like the experience. Additionally, no one had to stop the procedure before it ended, due to kinetic disease or any kind of other unpleasant sensations. Some patients claimed that they felt uncomfortable, disconnected, and unaware of the surroundings because they could not see what was happening around them. Similar observations were made by Ougradar et al. in their studies. Moreover, they observed that women were less impressed by VR and rated the VR experience lower than men did [30].

Even though the results are promising, there is definitely a need for further research. It should be performed on a larger study group and should implement some other audiovisual materials that are more interactive and more immersive. The current limitations of this study were the small sample size, lack of variety in the type of audiovisual content, lack of personal preferences in choosing the VR distraction, limited time of the procedure up to 30 min, and the large size of the headset, which made it bulky and not well-adjusted especially for women. Although the study used allocation concealment, it was not double-blind, which may have affected the outcome. Important for the results may also be that types of dental procedures were not equally distributed among the groups of patients. It is also worth mentioning that some of the initially recruited patients were excluded from the study only at the data analysis stage, based on the results of Hospital Anxiety and Depression Scale (HADS), which should also be considered a limitation. Furthermore, the poor quality of headphones made it impossible to completely block outside noises. Moreover, VR should be tested in comparison to other distraction methods like music or television as well as during some more complex dental procedures, e.g., surgeries or endodontic treatments, where anxiety control is more challenging.

## 5. Conclusions

The presented results, although promising, are just an introduction to the complex topic of virtual reality distraction among adult dental patients. This study showed that VR distraction can be a beneficial method for reducing dental anxiety. Psychophysiological parameters like heart rate and stress levels were significantly lower in patients who experienced VR. Moreover, the self-reported anxiety levels (based on STAI-X1) were also significantly lower for those patients who underwent VR distraction compared to the control group. Despite its limitations, the present study showed that VR can be used in dental offices to reduce stress and anxiety during treatment.

## Figures and Tables

**Table 1:** Sociodemographic characteristics of the participants included in the study.

	Group	Study Group (Total) *n* = 80 (100%)	Group 1 (VR1) *n* = 27	Group 2 (VR2) *n* = 25	Group 3 (Control) *n* = 28
	Age	Mean 36.1 years SD 12.61	Mean 39 years SD 12.47	Mean 31.2 years SD 12.42	Mean 37.8 years SD 12.01
Gender	Males	36 (45%)	12 (44.4%)	11 (44%)	13 (46.4%)
	Females	44 (55%)	15 (55.6%)	14 (56%)	15 (53.6%)
Place of residence	Country	36 (45%)	11(40.74%)	9 (36%)	16 (54.14%)
	Town less than 50 k	37 (46.25%)	12 (44.44%)	13 (52%)	12 (42.86%)
	City up to 100 k	3 (3.75%)	2 (7.41%)	1 (4%)	0 (0.0%)
	City over 100 k	4 (5%)	2 (7.41%)	2 (8%)	0 (0.0%)
Education	Professional	8 (10%)	3 (11.11%)	2 (8%)	3 (10.71%)
	Secondary	36 (45%)	9 (33.33%)	12 (48%)	15 (53.57%)
	Higher	36 (45%)	15 (55.56%)	11 (44%)	10 (35.71%)
Job status	Student	12 (15%)	3 (11.11%)	6 (24%)	3 (10.71)
	Employment contract	63 (78.75%)	23 (85.19%)	19 (76%)	21 (75%)
	Pension	2 (2.5%)	0 (0.0%)	0 (0.0%)	2 (7.14%)
	No job	3 (3.75%)	1 (3.7%)	0 (0.0%)	2 (7.14%)
Marital status	Marriage	41 (51.25%)	15 (55.56%)	9 (36%)	17 (60.71%)
	Informal relationship	17 (21.25%)	7 (25.93%)	6 (24%)	4 (14.19%)
	Single	22 (27.5%)	5 (18.52%)	10 (40%)	7 (25%)
Chronic conditions	Yes	11 (13.75%)	3 (11.11%)	1 (4%)	7 (25%)
	No	69 (86.25%)	24 (88.89%)	24 (96%)	21 (75%)
Psychiatric/psychological problems	Yes	4 (5%)	2 (7.41%)	2 (8%)	0 (0.0%)
	No	76 (95%)	25 (92.59%)	23 (92%)	28 (100%)
Chronic medication	Yes	13 (16.25%)	8 (29.66%)	1 (4%)	4 (14.29%)
	No	67 (83.75%)	19 (70.37%)	24 (96%)	24 (85.71%)

**Table 2:** Differences in psychological parameters in the VR1 group.

	At the Beginning		After 15 Min		At the End					
Dependent Variable	M	SD	M	SD	M	SD	F	*df*	*p*	η^2^
Heart rate	71.00	9.60	69.22	9.38	67.63	8.66	6.19	2	0.004	0.19
Saturation	97.67	2.50	96.89	3.00	97.11	2.83	1.86	2	0.166	0.07
Stress	0.14	0.09	0.17	0.16	0.13	0.11	2.00	2	0.145	0.07

**Table 3:** Differences in psychological parameters in the control group.

	At the Beginning		After 15 Min		At the End					
Dependent Variable	M	SD	M	SD	M	SD	F	*df*	*p*	η^2^
HR	74.86	13.58	74.39	11.76	75.18	11.70	0.10	2	0.903	0.004
Saturation	97.67	2.50	96.89	3.00	97.11	2.83	1.86	2	0.166	0.067
Stress	0.15	0.09	0.20	0.12	0.21	0.10	4.45	2	0.02	0.15

**Table 4:** Differences in psychological parameters between VR1 and the control group.

	VR1 Group		Control Group					
Dependent Variable	M	SD	M	SD	*t*	*df*	*p*	d
HR—beginning	71.00	9.60	74.86	13.58	−1.22	48.7	0.114	0.32
HR—15 min	69.22	9.38	74.39	11.76	−1.80	53	0.039	0.49
HR—end	67.63	8.66	75.18	11.70	−2.71	53	0.005	0.73
SAT—beginning	97.67	2.50	96.57	2.33	1.68	53	0.049	0.45
SAT—15 min	96.89	3.00	97.25	2.26	−0.50	53	0.615	0.14
SAT—end	97.11	2.83	97.04	2.12	0.11	53	0.456	0.03
Stress—beginning	0.14	0.09	0.15	0.09	−0.76	53	0.451	0.21
Stress—15 min	0.17	0.16	0.20	0.12	−0.71	53	0.480	0.20
Stress—end	0.13	0.11	0.22	1.10	−2.91	53	0.003	0.80

**Table 5:** Differences in psychological parameters—STAI—in the VR2 group, first vs. second visit.

	Measurement 1		Measurement 2					
Dependent Variable	M	SD	M	SD	*t*	*df*	*p*	d
STAI	29.56	7.80	27.12	5.41	2.29	24	0.02	0.46

**Table 6:** Differences in psychological parameters—STAI—in the control group, first vs. second visit.

	Measurement 1		Measurement 2					
Dependent Variable	M	SD	M	SD	*t*	*df*	*p*	d
STAI	29.32	7.65	27.93	6.91	1.09	27	0.14	0.21

**Table 7:** Differences in psychological parameters—STAI—between the VR2 and control groups.

	VR2 Group		Control Group					
Dependent Variable	M	SD	M	SD	*t*	*df*	*p*	d
STAI	27.12	5.41	27.93	6.90	−0.47	51	0.32	0.13

## Data Availability

The data presented in this study are available on request from the corresponding author.

## Supplementary Materials

The following supporting information can be downloaded at: https://www.mdpi.com/article/10.3390/jcm13226832/s1, Table S1: Results of the χ^2^ difference test for sociodemographic variables regarding sample sizes across all subgroups (VR1, VR2, Control).
