# Supplementary material for: An Attempt to Use Virtual Reality as a Tool to Reduce Patient Anxiety During Dental Treatment
Source: J Clin Med. 2024 Nov 13;13(22):6832. doi: 10.3390/jcm13226832 (PMC11594890; doi:10.3390/jcm13226832)
Supplement: Supplementary file 1 [file jcm-13-06832-s001.zip › jcm-3258733-supplementary.pdf]

Additional analyses for manuscript: An attempt to use virtual reality as a tool to reduce patient anxiety during dental treatment.

1. VR1 Group

Objective: Calculate the significance of differences in heart rate, saturation, and stress level during the first measurement (Visit 1): at the beginning, in the middle (after 15 minutes), and at the end.

H1. Heart Rate To determine if VR usage affects heart rate changes, a one-way repeated measures ANOVA was conducted. The within-subject factor measured at three levels was the time (beginning, after 15 minutes, and end of the visit). Heart rate, measured using a pulse oximeter (NOVAMA®), served as the dependent variable. The ANOVA revealed a significant main effect of time, $F(2,52)=6.19$; $p<0.01$; $\eta^2=0.19$. A post hoc analysis (Bonferroni correction for multiple comparisons) showed that the heart rate at the end ($M=67.63$; $SD=9.60$) was significantly lower than at the beginning ($M=71.00$; $SD=8.66$), with significance at $p<0.05$. There were no significant differences between the heart rate at the beginning, after 15 minutes, and between the latter two times. The analysis suggests a significant reduction in heart rate at the end compared to the beginning but no significant drop after 15 minutes compared to the start.

H2. Saturation No significant differences.

H3. Stress No significant differences.

Objective: Calculate the significance of differences in heart rate, saturation, and stress level during the second measurement (Visit 2): at the beginning, in the middle (after 15 minutes), and at the end.

H1. Heart Rate No significant differences.

H2. Saturation No significant differences.

H3. Stress No significant differences.

Objective: Test the significance of differences in STAI (State-Trait Anxiety Inventory) between measurements 1 and 2 (Visits 1 and 2).

Result: Although anxiety levels were lower in the first measurement, this difference was not statistically significant.

Objective: Test the significance of differences in MDAS (Modified Dental Anxiety Scale) between measurements 1 and 2 (Visits 1 and 2).

Result: There was a decrease, but it lacked statistical significance ($p=0.067$, $p>0.05$), meaning the hypothesis could not be confirmed.

2. Group VR2

Objective: Calculate the significance of differences in heart rate, saturation, and stress level during the first measurement (Visit 1): at the beginning, in the middle (after 15 minutes), and at the end.

H1. Heart Rate No significant differences.

H2. Saturation No significant differences.

H3. Stress No significant differences.

Objective: Calculate the significance of differences in heart rate, saturation, and stress level during the second measurement (Visit 2): at the beginning, in the middle (after 15 minutes), and at the end.

H1. Heart Rate No significant differences.

H2. Saturation No significant differences.

H3. Stress No significant differences.

Objective: Test the significance of differences in STAI between measurements 1 and 2 (Visits 1 and 2).

Result: A paired-samples t-test revealed that the mean anxiety level during Visit 2 in the VR2 group (M=27.12; SD=5.41) was significantly lower than during Visit 1 (M=29.56; SD=7.80), t(24)=2.29; p<0.05. Cohen's d=0.46 indicates a weak relationship between measurement time and anxiety level.

Objective: Test the significance of differences in MDAS between measurements 1 and 2 (Visits 1 and 2).

Result: The dependant-samples t-test confirmed that the MDAS anxiety level in the VR2 group was the same during both visits.

Objective: **:** Test the significance of differences in HR, saturation and stress in VR2 (between visits)

a. At the beginning - measurements 1 and 2 (Visits 1 and 2)
    - **H1. Heart Rate** No significant differences.
    - **H2. Saturation** No significant differences.
    - **H3. Stress** No significant differences.
b. After 15 min - measurements 1 and 2 (Visits 1 and 2)
    - **H1. Heart Rate** No significant differences.
    - **H2. Saturation** No significant differences.
    - **H3. Stress** No significant differences.
c. At the end - measurements 1 and 2 (Visits 1 and 2)
    - **H1. Heart Rate** No significant differences.
    - **H2. Saturation** No significant differences.
    - **H3. Stress** No significant differences.

3. Control Group

Objective: Calculate the significance of differences in heart rate, saturation, and stress level during the first measurement (Visit 1): at the beginning, in the middle (after 15 minutes), and at the end.

H1. Heart Rate No significant differences.

H2. Saturation No significant differences.

H3. Stress No significant differences.

Objective: Calculate the significance of differences in heart rate, saturation, and stress level during the second measurement (Visit 2): at the beginning, in the middle (after 15 minutes), and at the end.

H1. Heart Rate: No significant differences.

H2. Oxygen Saturation: No significant differences.

H3. Stress: No significant differences.

A statistical issue arose here: the assumption of sphericity (a necessary condition for this analysis), measured using Mauchly's test, was not met (p-value exceeded 0.05). Therefore, a repeated measures ANOVA with the Greenhouse-Geisser correction was used. While the significance level for sphericity was $p=0.03$, adjusting with the correction yielded $p=0.42$. This impacted the Bonferroni-corrected post hoc test results, which, despite (theoretically) indicating significance for the overall analysis (F), showed no significant differences between individual measurements. In other words, when the model's significance hovers around $p=0.05$, the observed differences are likely artifacts rather than real differences.

Objective: Test the significance of differences in STAI (State-Trait Anxiety Inventory) between measurements 1 and 2 (Visits 1 and 2).

Results: A dependant-samples t-test was conducted to assess the difference in STAI levels between the first and second visits. The analysis showed that the mean anxiety level in the control group during the second visit was the same as during the first visit, $t(27)=1.09$; $p>0.05$.

Objective: Test the significance of differences in MDAS (Modified Dental Anxiety Scale) between measurements 1 and 2 (Visits 1 and 2).

Results: A dependant-samples t-test was conducted to assess the difference in MDAS levels between the first and second visits. The analysis showed that the mean anxiety level in the control group during the second visit was the same as during the first visit, $t(27)=0.42$; $p>0.05$.

4. Comparison between VR1 and Control during first measurement (visit 1).

Objective: Test the significance of differences for Heart Rate, Saturation, and Stress levels in the first measurement (visit 1)

a. at the beginning (VR1 vs C)

- **H1. Heart Rate**: To evaluate the difference in heart rate at the beginning of the first measurement between the VR1 group and the control group, an independent samples t-test was conducted. The test revealed that the average heart rate in the VR1 group was the same as in the control group, $t(1,48) = -1.21$; $p > 0.05$.
- **H2. Saturation**: An independent samples t-test was performed to assess the difference in oxygen saturation between the VR1 group and the control group at the beginning of the first measurement. The analysis showed that the average saturation in the VR1 group was higher ($M=97.67$; $SD=2.49$) than in the control group ($M=96.57$; $SD=2.33$), $t(1,53) = 1.68$; $p < 0.05$. Cohen's $d = 0.46$ indicates a weak relationship between group type and saturation.

- **H3. Stress**: No significant differences were found.

b. **After 15 min (VR1 vs C)**

- **H1. Heart Rate**: To examine the difference in heart rate after 15 minutes in the VR1 group and the control group, an independent samples t-test was conducted. The test revealed that the average heart rate in the VR1 group (M=69.22; SD=9.38) was lower than in the control group (M=74.39; SD=11.76), $t(1,53) = -1.80$; $p < 0.05$. Cohen's d = 0.45 indicates a weak association between group type and heart rate.
- **H2. Saturation**: No significant differences were found.
- **H3. Stress**: No significant differences were found.

c. **at the end (VR1 vs C)**

- **H1. Heart Rate**: To assess the difference in heart rate at the end of the first measurement between the VR1 group and the control group, an independent samples t-test was conducted. The analysis showed that the average heart rate in the VR1 group (M=67.63; SD=8.66) was lower than in the control group (M=75.18; SD=11.70), $t(1,53) = -2.71$; $p < 0.01$. Cohen's d = 1.00 suggests a strong relationship between group type and heart rate.
- **H2. Saturation**: No significant differences were found.
- **H3. Stress**: To evaluate the difference in stress levels at the end of the first measurement between the VR1 group and the control group, an independent samples t-test was conducted. The test indicated that the average stress level in the VR1 group (M=0.13; SD=0.11) was lower than in the control group (M=0.22; SD=0.10), $t(1,53) = -2.91$; $p < 0.01$. Cohen's d = 1.16 indicates a strong association between group type and stress level.

Objective: Test the significance of differences in STAI (State-Trait Anxiety Inventory) between VR1 and Control in measurements 1 (visit 1).

Result: No significant differences were found.

5. Comparison between VR2 and Control during second measurement (visit 2).

Objective: Test the significance of differences for Heart Rate, Saturation, and Stress levels in the second measurement (visit 2)

a. **at the beginning (VR1 vs C)**
    - **H1. Heart Rate** No significant differences.
    - **H2. Saturation** No significant differences.
    - **H3. Stress** No significant differences.

b. **After 15 min (VR1 vs C)**
    - **H1. Heart Rate** No significant differences.
    - **H2. Saturation** No significant differences.
    - **H3. Stress**: To examine the difference in stress levels after 15 minutes in the second measurement between the VR2 group and the control group, an independent samples t-test was conducted. The test revealed that the mean stress level in the VR2 group (M=0.16; SD=0.09) was lower than in the control

group (M=0.22; SD=0.14), t(1,53) = -1.91; p < 0.01. Cohen's d = 0.50 indicates a moderate relationship between group type and stress level.

c. **at the end (VR1 vs C)**

- **H1. Heart Rate** To assess the difference in heart rate at the end of the second measurement between the VR2 group and the control group, an independent samples t-test was conducted. The test showed that the mean heart rate in the VR2 group (M=75.40; SD=10.00) was lower than in the control group (M=70.68; SD=10.09), t(1,53) = -1.71; p < 0.01. Cohen's d = 0.40 indicates a weak association between group type and heart rate.
- **H2. Saturation** No significant differences.
- **H3. Stress** No significant differences.

Objective: Test the significance of differences in STAI (State-Trait Anxiety Inventory) between VR2 and Control in measurements 2 (visit 2).

Result: No significant differences were found.

Significance of differences in sample sizes across sociodemographic variables.

To compare groups VR1 (n=27), VR2 (n=25), and the control group (n=28) in order to verify the significance of differences in sample sizes across all sociodemographic variables, as well as chronic conditions and medications used by the participants, a non-parametric Pearson chi-square test was applied. The test results did not reveal any significant differences in the number of individuals in each group concerning all sociodemographic data, as well as the occurrence of chronic and mental illnesses. A significant difference in sample sizes was found only in relation to chronic medications used by the participants ($\chi^2_{(2)}=6.39,\ p<0.05$), indicating statistically significant disparities in this variable among the VR1, VR2, and control groups ($n_{VR1}=8$, $n_{VR2}=1$, $n_K=4$). The following table presents the Pearson chi-square test results for all variables from the demographic survey.

Table S1. Results of the χ^2 difference test for sociodemographic variables regarding sample sizes across all subgroups (VR1, VR2, control).

Variable	Value χ^2 test	df	Asymptotic Significance (p)
Gender	0.037	2	0.98
Place of residence	5.92	6	0.43
Education	2.68	4	0.61
Job status	7.69	6	0.26
Marital status	5.00	4	0.29
Chronic conditions	5.15	2	0.08
Psychiatric/psychological problems	2.28	2	0.32
Chronic medication	6.39	2	0.04*

* Significant difference (p<0.05)
